# Complete chloroplast genome of *Prunus fruticosa* and its implications for the phylogenetic position within *Prunus sensulato* (Rosaceae)

**DOI:** 10.1080/23802359.2020.1831993

**Published:** 2020-10-27

**Authors:** Yu-Xin Yang, Mi-Hui Tian, Xin-Hong Liu, Yue-ling Li, Zhong-Shuai Sun

**Affiliations:** a College of Life Sciences, Taizhou University, Taizhou, China; b Zhejiang Academy of Forestry, Hangzhou, China; c Zhejiang Provincial Key Laboratory of Plant Evolutionary Ecology and Conservation, Taizhou University, Taizhou, China

**Keywords:** *Prunus fruticose*, *Prunus sensu lato*, chloroplast genome, phylogenomics

## Abstract

*Prunus fruticosa* is a wild species of *Prunus* distributed across the central Eurasia. Here, we reported the complete chloroplast (cp) genome of *P. fruticosa* (GenBank accession number: MT916286). The cp genome was 158,217 bp long, with a large single-copy region (LSC) of 86,322 bp and a small single-copy region (SSC) of 19,153 bp separated by a pair of inverted repeats (IRs) of 26,371 bp. It encodes 129 genes, including 84 protein-coding genes, 37 tRNA genes, and 8 ribosomal RNA genes. We also reconstructed the phylogeny of *Prunus sensu lato* using maximum likelihood (ML) method, including our data and previously reported cp genomes of related taxa. The phylogenetic analysis indicated that *P. fruticosa* is closely related with *Prunus avium*.

*Prunus fruticosa* Pall. is a wild species that is widespread in a major part of Central Europe, Eastern Central Europe, the Balkan Peninsula, Eastern Europe, Apennine Peninsula, the Caucasus, West Siberia, and Central and Northern Asia (Hedrick 1915; Webb 1968; Shishkin and Yuzeqchuk 1971). *P. fruticosa* together with *P. avium* L. (sweet cherry) and *P. cerasus* L. (sour cherry) constitute Eucerasus section (Dirlewanger et al. 2007), and studies have shown that *P. cerasus* arose from natural hybridization between *P. fruticosa* and *P. avium* several times through history (Iezzoni 2005; Macková et al. 2018). However, the phylogenetic relationships between *P. fruticose* and its related species are debated due to the still unsolved phylogenetic system of *Prunus sensu lato* (Rosaceae) (Shi et al. 2013; Chin et al. 2014). By taking advantages of next-generation sequencing technologies that efficiently provide the chloroplast (cp) genomic resources of our interested species, we can rapidly access the abundant genetic information for phylogenetic research and conservation genetics (Li et al. 2017; Liu et al. 2017). Therefore, we sequenced the whole chloroplast genome of *P. fruticosa* to elucidate its phylogenetic relationship with other species in *Prunus sensu lato*.

Total genomic DNA was extracted from silica-dried leaves collected from Heilongjiang Forest Botanical Garden (Harbin, Heilongjiang, China) using a modified CTAB method (Doyle and Doyle 1987). A voucher specimen (Sun_HLJ) was collected and deposited in the Herbarium of Taizhou University. DNA libraries preparation and pair-end reads sequencing were performed on the Illumina NovaSeq 6000 platform. The cp genome was assembled via NOVOPlasty (Dierckxsens et al. 2017), using the *Prunus rufa* cp genome (MN648456; Li et al. 2020) as a reference. Gene annotation was performed via the online program Dual Organellar Genome Annotator (DOGMA; Wyman et al. 2004). Geneious R11 (Biomatters Ltd., Auckland, New Zealand) was used for inspecting the cp genome structure.

The complete cp genome of *P. fruticosa* (GenBank accession MT916286) was 1,58,217 bp long consisting of a pair of inverted repeat regions (IRs with 26,371 bp) divided by two single-copy regions (LSC with 86,322 bp; SSC with 19,153 bp). The overall GC contents of the total length, LSC, SSC, and IR regions were 36.6, 34.4, 30.0, and 42.5%, respectively. The genome contained a total of 129 genes, including 84 protein-coding genes, 37 tRNA genes and 8 rRNA genes.

We used a total of 22 additional complete cp genomes of the *Prunus sensu lato* species to clarify the phylogenetic position of *P. fruticosa*. *Prunus serotina* Ehrh. (NC036133) and *P. padus* L. (NC026982) in Subg. *Padus* were used as the outgroup. We reconstructed a phylogeny employing the GTR + G model and 1000 bootstrap replicates under the maximum-likelihood (ML) inference in RAxML-HPC v.8.2.10 on the CIPRES cluster (Miller et al. 2010). The ML tree (Figure 1) was consistent with the most recent phylogenetic study on *Prunus sensu lato* (Shi et al. 2013; Chin et al. 2014). *P. fruticosa* exhibited the closest relationship with *Prunus avium* L.

**Figure 1.**
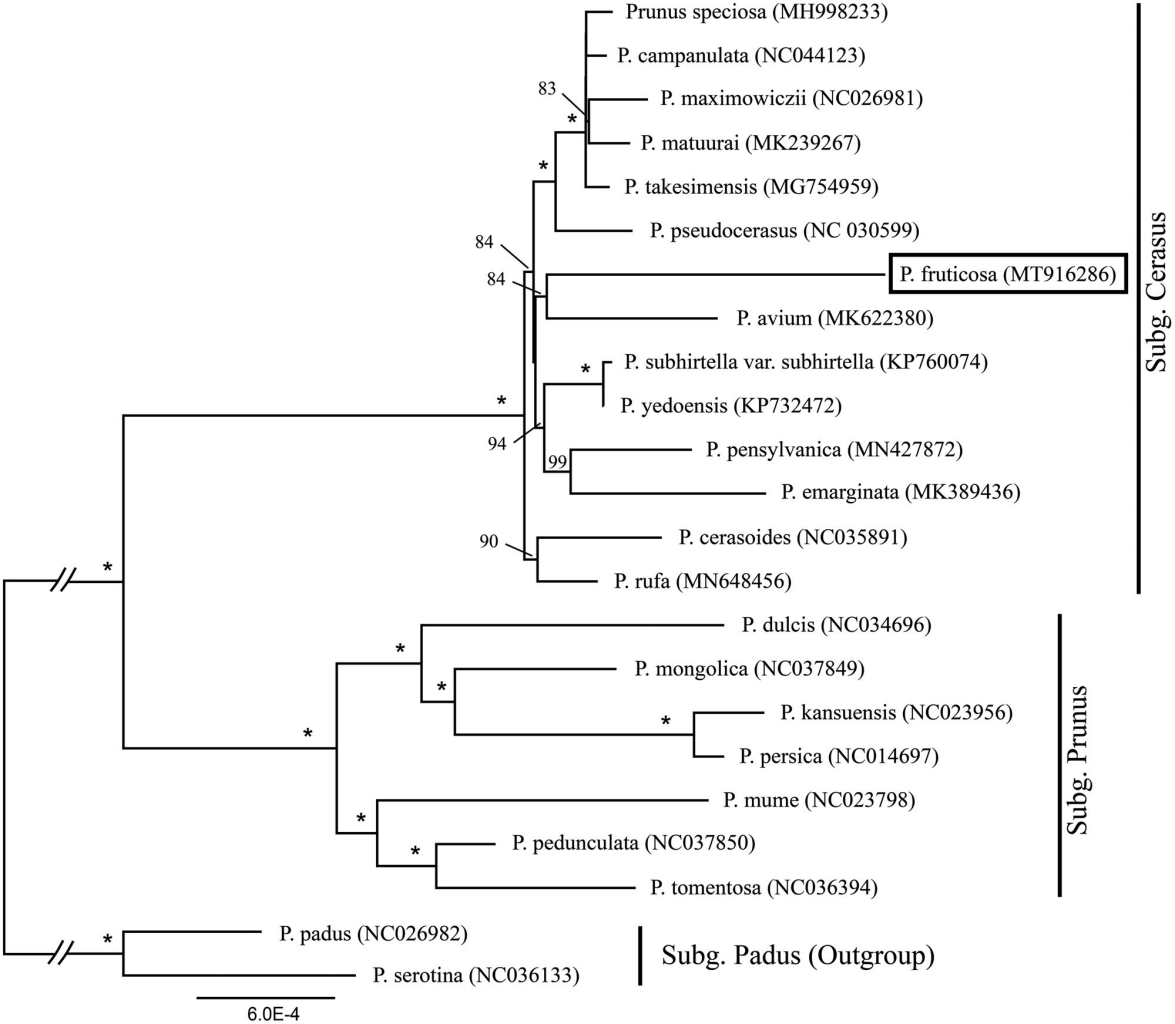
Phylogenetic tree reconstruction of 23 taxa of *Prunus sensu lato* using ML method. Relative branch lengths are indicated. Support values above the branches are ML bootstrap support; “*” indicates 100% support values.

## Data Availability

The data that support the findings of this study are openly available in GenBank of NCBI at https://www.ncbi.nlm.nih.gov, reference number MT916286. The raw sequencing reads used in this study was deposited in the Sequence Read Archive (SRA) under accession number PRJNA659616.
